# Optimization of the Filler-and-Binder Mixing Ratio for Enhanced Mechanical Strength of Carbon–Carbon Composites

**DOI:** 10.3390/ma16114084

**Published:** 2023-05-30

**Authors:** Ji-Hong Kim, Hye-In Hwang, Ji-Sun Im

**Affiliations:** 1 Hydrogen & C1 Gas Research Center, Korea Research Institute of Chemical Technology (KRICT), Daejeon 34114, Republic of Korea; pic10@krict.re.kr (J.-H.K.); gpdls474@krict.re.kr (H.-I.H.); 2 Department of Chemical Engineering and Applied Chemistry, Chungnam National University, Daejeon 34134, Republic of Korea; 3 Advanced Materials and Chemical Engineering, University of Science and Technology (UST), Daejeon 34113, Republic of Korea

**Keywords:** binder mixing ratio, mechanical strength, binder pitch, carbon–carbon composite, synthetic graphite, coke–pitch interaction

## Abstract

In this paper, a method for optimizing the mixing ratio of filler coke and binder for high-strength carbon–carbon composites is proposed. Particle size distribution, specific surface area, and true density were analyzed to characterize the filler properties. The optimum binder mixing ratio was experimentally determined based on the filler properties. As the filler particle size was decreased, a higher binder mixing ratio was required to enhance the mechanical strength of the composite. When the d_50_ particle size of the filler was 62.13 and 27.10 µm, the required binder mixing ratios were 25 and 30 vol.%, respectively. From this result, the interaction index, which quantifies the interaction between the coke and binder during carbonization, was deduced. The interaction index had a higher correlation coefficient with the compressive strength than that of the porosity. Therefore, the interaction index can be used in predicting the mechanical strength of carbon blocks and optimizing their binder mixing ratios. Furthermore, as it is calculated from the carbonization of blocks without additional analysis, the interaction index can be easily used in industrial applications.

## 1. Introduction

Carbon–carbon (C–C) composites, such as isotropic graphite and graphite electrode, have a bulk structure and are applied in the steelmaking, semiconductor, nuclear energy, and aerospace industries [1,2,3,4,5,6]. For their applications as structural materials, the mechanical strength, namely the compressive and flexural strength, of C–C composites is essential [5,6,7,8]. Patrick and Albert reported that the mechanical strength of carbon materials follows the Knudsen-type exponential equation, whereby mechanical strength converges to its theoretical value with reducing porosity [9,10].

C–C composites are prepared by a series of processes, namely the mixing of the filler and binder, pressing or extrusion, and thermal treatment, including carbonization and graphitization [1,3,5,11,12]. Needle and regular coke are used as the filler of C–C composites, whereas the pitch, which has similar properties to the coke during carbonization, is used as the binder [5,13,14]. In particular, the binder pitch is polymerized by dehydrogenation, aromatization, and condensation during carbonization, which is accompanied by cracking as a side reaction [12,13,14,15,16]. Light molecules formed by the pitch cracking are volatilized during carbonization, which affects the porosity of the composites [1]. Therefore, the amount of binder pitch should be suitably controlled, and the mixing ratio of the filler and binder is a main variable for the strength of C–C composites [12,15].

The affinity of the filler and binder is also an essential factor in the mechanical strength of C–C composites [2,5,17,18]. The crystallinity, particle size distribution, and surface roughness of coke influences its affinity with the binder pitch, which is referred to as wettability [2,17,18]. Mechanical strength can be changed based on the characteristics of filler particles, which have a high affinity with the binder pitch. Therefore, the aforementioned properties of the filler particles should be considered in optimizing the filler-and-binder mixing ratio.

This paper discusses the optimization method of the mixing ratio of the filler coke and binder pitch for high-strength C–C composites, considering the properties of the filler particles, namely the average particle size, particle size distribution, specific surface area, and pore structure. The particle properties were controlled by pulverizing coke of various particle sizes. The prepared cokes were mixed with 10–40 vol.% binder and the carbon block was prepared by hot-pressing. The apparent density, porosity, and compressive strength of the prepared blocks were investigated. Subsequently, the optimum conditions to prepare high-strength C–C composites were determined from the comparative analysis of the block characteristics. The interaction index was suggested for optimizing the filler-and-binder mixing ratio, thereby predicting and enhancing the strength of the blocks.

## 2. Experimental Section

### 2.1. Materials

Regular coke (POSCO MC Materials, Gwangyang, Republic of Korea) was used as the filler for the carbon block. The calcination of regular coke at 1300 °C resulted in its lower crystallinity than that of graphite. The X-ray diffraction pattern of regular coke is shown in Figure S1. A coal-based pitch was used as the binder (Handan Jinghao Chemical Co., Ltd., Handan, China). The softening point and coking value of the binder were 103.4 °C and 34.3 wt.%, respectively. The detailed properties of the coke and pitch used in this study are listed in Table 1.

### 2.2. Experiments

#### 2.2.1. Preparation of the Filler Coke

In a 700 mL milling chamber with 200 g SUS ball (Φ5 mm), 200 g lump coke was added. Subsequently, the chamber was vertically shaken at 700 rpm for 40 min to pulverize the sample. The prepared powder coke was sieved with different meshes of 200–270 (74–53 µm), 270–400 (53–37 µm), and 400–800 (37–15 µm); the resulting fillers are referred to as C60, C40, and C20 (based on the d_50_ particle size), respectively.

#### 2.2.2. Preparation of the Coke/Binder Mixture

In this study, the coke/binder mixing ratio was optimized based on the effect of the amount of binder pitch. In particular, the binder in C–C composites or synthetic graphite is responsible for the attachment of the filler particles. Therefore, the coke/binder mixing was considered based on the volumetric ratio. The mixing ratio was converted to the weight format from the volumetric ratio using the true density, as shown in Table S1.

First, 10 g coke/binder mixture was kneaded with 10 mL tetrahydrofuran (THF) at 75 °C for 60 min. The mixture was kept in a convection oven at 80 °C for 24 h to remove the residual THF. In a cylinder mold with a diameter of 10 mm, 1 g of the prepared mixture was charged and hot-pressed at 110 °C for 10 min under 33.34 MPa (340 kgf/cm^2^). The carbon block was prepared by the carbonization of the block at 900 °C for 60 min.

### 2.3. Characterization

#### 2.3.1. Pulverized Coke Properties

The field-emission scanning electron microscopy (FE-SEM) micrographs (TESCAN, Brno, Czech Republic) taken with the acceleration voltage of an electron beam of 10 kV and magnification of 200–5000 were used to observe the size and shape of pulverized coke particles. Particle size distribution was used to measure the size of the coke particles and was analyzed by water dispersion under tip sonication. Tap density was considered to estimate the packing density of pulverized coke particles using BT-301 (BETTERSIZE, Dandong, China). Tap density was calculated from the volume measured after tapping 3000 times at 300 taps/min. The specific surface area of the pulverized coke was characterized by N_2_ adsorption at −196 °C using an ASAP 2420 (Micrometrics, Norcross, GA, USA) and calculated using the Brunauer−Emmett−Teller (BET) equation.

#### 2.3.2. Density

The apparent and true density of the prepared carbon blocks were investigated. The apparent density was observed using Shinko Denshi Co., Ltd. (Tokyo, Japan) DME-220E density meter through an underwater replacement method (JIS Z8807). The true density was investigated using a Micromeritics (Norcross, GA, USA) AccuPyc II 1340 Pycnometer under helium gas.

The volume for apparent density was measured by the underwater replacement method and was assumed to include pores in the carbon block as the block is a hydrophobic material. The volume for true density was measured under helium gas, so it was assumed to exclude pores [19,20]. The porosity of the carbon block was calculated using Equation (1):

(1) $$Porosity(\%)=\left(1-\frac{Apparent\;density}{True\;density}\right)\times 100$$

#### 2.3.3. Mechanical Strength

The compressive strength was investigated to estimate the mechanical strength of the carbon block. The carbon block was formed into a cylinder with a diameter of 10 mm. The compressive strength was obtained according to the test condition of ASTM C0695-15. Compression tests were conducted on the Instron (Norwood, MA, USA) Series IX automated materials testing system with a load capacity of 3000 kgf. The rate of crosshead movement was applied at a rate of 1.3 mm/min.

## 3. Results and Discussion

### 3.1. Characteristics of the Pulverized Regular Coke

**Figure 1:**
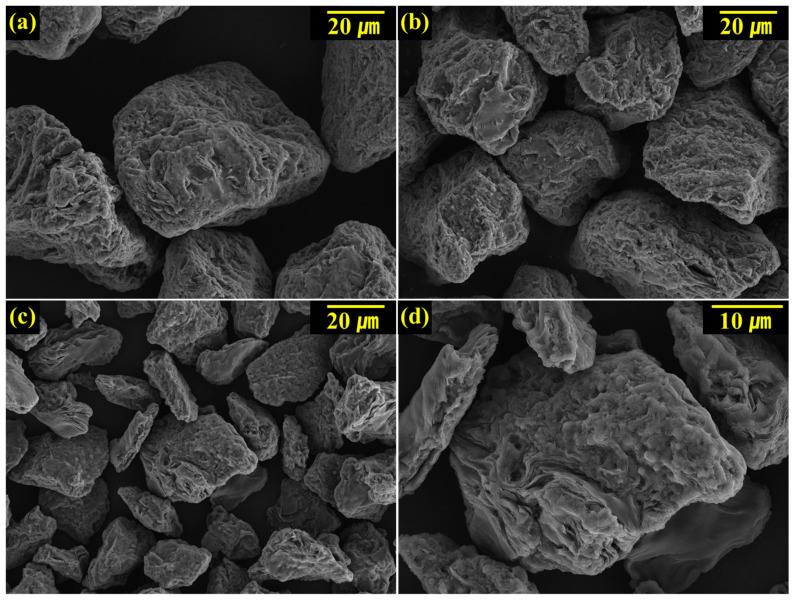
SEM images of the size-controlled coke particles using (**a**) C60, (**b**) C40, (**c**) C20, and (**d**) C20 fillers (magnification 5000×).

In this study, coke with various filler sizes was used to optimize the binder ratio of carbon blocks based on the filler characteristics. Figure 1 shows the FE-SEM images of the pulverized coke particles. The particle sizes in Figure 1a,b, and c were 60, 40, and 20 µm, respectively. Although the particles have irregular shapes, their aspect ratios were approximately 1. Graphitic carbon has crystals with a layered structure, resulting in flake-type particles. In contrast, regular coke has an irregular orientation, as shown in Figure 1d, indicating that it can be pulverized to an irregular shape. This result is also observed in the X-ray diffraction pattern (Figure S1), in which regular coke has lower crystallinity than that of graphite or needle coke [21].

**Figure 2:**
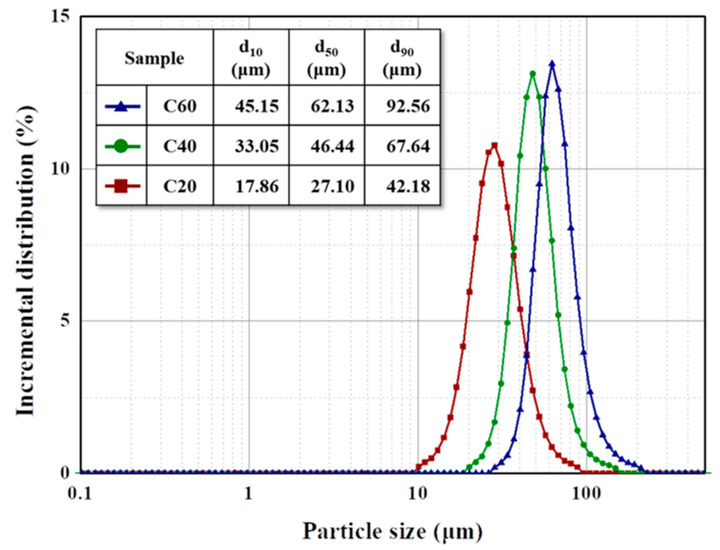
Particle size distribution of the prepared coke particles.

Particle size distribution was used to measure the size of the coke particles, as shown in Figure 2. As with the SEM images, the particle size d_50_ was controlled to 62.13, 46.44, and 27.10 µm for the C60, C40, and C20 fillers, respectively. The SPAN value, which represents the width of the particle size distribution, was calculated to compare the particle size distribution using Equation (2):

(2) $$SPAN=\frac{d_{90}-d_{10}}{d_{50}}$$

A higher SPAN value indicates a broad distribution. The calculated SPAN values, true density, tap density, and specific surface area of the coke particles are listed in Table 2. The SPAN values and average particle size exhibit a clear trend. The SPAN value is affected by the sieve size used for the filler classification. In particular, the SPAN value increased according to the gap of the used sieve size.

Tap density is mainly influenced by the SPAN value, whereby a higher SPAN value improves packing by removing the free volume [22,23]. However, in this study, tap density decreased with the decreasing particle size. When the particle size was less than 100 µm, the interparticle attractive forces, such as the van der Waals and attractive electrostatic forces, became more prominent than the gravity force, resulting in the agglomeration of the particles and the formation of voids inside the agglomerated coke with an irregular shape [3,4,24].

True density increased with the decrease in the particle size. As volume includes the closed pore inside the coke particle, which can open during pulverization, in the calculation of the true density, the true density slightly increased with the decreasing particle size [25]. The specific surface area is analyzed by the N_2_ adsorption and desorption isotherms, as shown in Figure S2. The surface area mainly increased by decreasing the particle size and increasing the surface roughness caused by the opened pores [25,26].

### 3.2. Effect of the Binder Ratio on the Properties of the Carbon Block

Pulverized samples, C60, C40, and C20, were mixed with binder pitch volume ratios of 10–40% to form the carbon block. For C40 or C20 with 10 vol.% binder, the ratio of the binder pitch was too low for the specific surface area, which resulted in the inability of the carbon block to maintain its bulk shape (unmoldable) and the formation of cracks and breakage, as shown in Figure S3a. In contrast, the binder pitch leaked out of the pressing mold with an excessive ratio of the binder pitch, as shown in Figure S3b. Therefore, the suitable binder ratio of 10–35 and 15–40 vol.% was determined to shape carbon blocks using C60 and C40 and C20 fillers, respectively.

**Figure 3:**
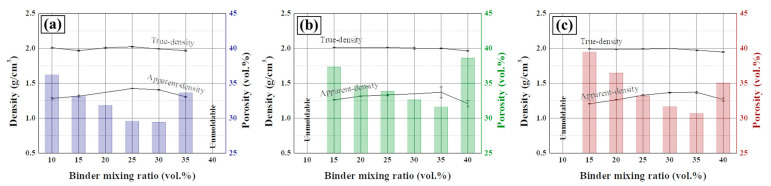
Effect of the binder mixing ratio on the true density, apparent density, and porosity of the prepared carbon blocks using (**a**) C60, (**b**) C40, and (**c**) C20 as the filler.

The density and porosity of carbon blocks prepared under the determined suitable conditions are presented in Figure 3. In Figure 3a, the true density was maintained at approximately 2.0 g/cm^3^. Thus, the carbonized pitch has a similar density to that of the regular coke. The apparent density varied with the binder mixing ratio, showing a maximum value with 25–30 vol.% binder ratios. Moreover, the porosity has an inverse relationship with the apparent density, which means that porosity is mainly influenced by changing the apparent density. Samples using C40 and C20 exhibit the same tendency as those using C60 (Figure 3b,c); however, their optimum binder ratio was higher at 35 vol.%. With a binder ratio of 15 vol.%, the porosity gradually increased to 33.10, 37.31, and 39.43% using C60, C40, and C20 fillers, respectively. This trend was maintained up to a binder ratio of 20 vol.%.

When the binder ratio was less than the optimum condition (in the binder-shortage condition), the porosity is influenced by the surface area of the filler coke. In particular, with a small surface area of the filler, binder demand decreased. Therefore, the porosity decreased with the increasing particle size with the same binder ratio. Particles with a high surface area require more binder under the binder-shortage and -excessive condition. However, this has different effects on the porosity. For example, with a 35 vol.% binder ratio, the porosity gradually decreased to 33.62%, 31.56%, and 30.60% with C60, C40, and C20 fillers, respectively, which differs from the binder-shortage condition.

The binder pitch is responsible for the attachment between the surface of the filler particles and filling the void space between the filler particles. The binder requirement to attach to the particle surface decreased with the increasing particle size. That is, the role of filling the void space increased with the increasing particle size in the same binder ratio. However, when the optimal binder ratio is exceeded, the binder ratio can satisfy its roles, and a surplus pitch is obtained. This surplus pitch causes cracks by the expansion in the carbon block because of its volatilization during thermal treatment. The volatilized pitch is released into the space between the particles, whereby smaller particle size provides more pathways in the same volume. Therefore, the smaller particle size allows flexibility of the surplus binder in the binder-excessive conditions.

**Figure 4:**
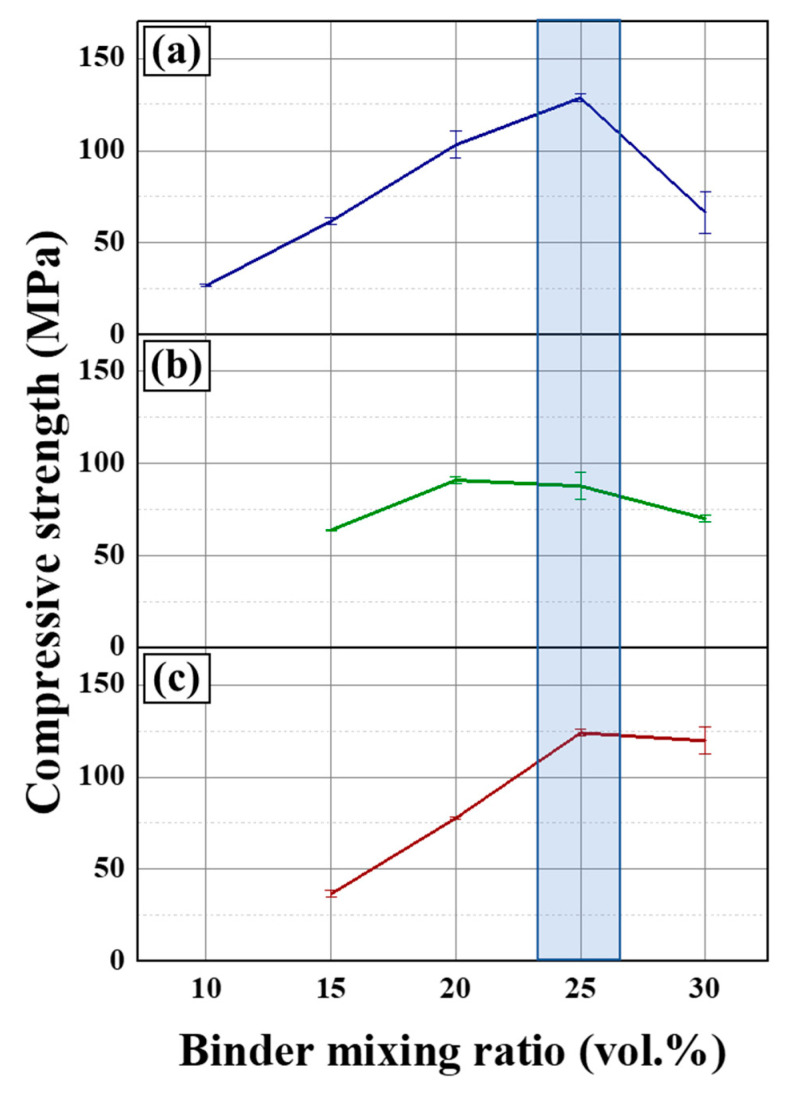
Compressive strength of the prepared blocks using (**a**) C60, (**b**) C40, and (**c**) C20 as the filler.

The mechanical strength of the prepared carbon blocks is shown in Figure 4. The maximum compressive strength of the prepared samples was noted with a 25 vol.% binder ratio for all particle sizes. With a 20 vol.% binder ratio, the compressive strength decreased to 103.02, 90.94, and 77.78 MPa as the particle size of the filler was decreased (C60, C40, and C20, respectively). However, with a 30 vol.% binder ratio, strength increased to 66.32, 70.12, and 119.72 MPa using the C60, C40, and C20 filler, respectively. With the optimum binder ratio of 25 vol.%, the mechanical strength of the prepared carbon blocks displayed a similar tendency to the porosity. For example, the binder-excessive condition, C20, which has the smallest particle size, has surplus binder. Meanwhile, with the increase of the binder mixing ratio from 25 to 30 vol.%, the mechanical strength using C60 rapidly decreased from 128.61 to 66.32 MPa, whereas it slightly decreased from 124.04 to 119.72 MPa using C20. Nonetheless, the trends of the porosity and mechanical strength have notable differences. When the particle size decreased with the same binder ratio, the porosity increased in the binder-shortage condition, whereas the mechanical strength did not vary considerably.

As discussed in Section 3.1, based on the pore opening, the particle roughness increased with the reduced particle size. Thus, more surface roughness provides a higher surface area for the interaction between the filler coke and binder molecules. Moreover, complex structures on the filler surface with the pore opening can cause an anchor effect between the filler and binder [25,26]. Consequently, the mechanical strength can be increased using smaller particles as the filler. The porosity also increased with the smaller particles, which is an incompatible tendency because the mechanical strength is enhanced by decreasing the porosity in the Knudsen equation. This means that the compressive strength has more variables than the porosity [27]. Therefore, the optimal factor of the binder mixture ratio to improve the mechanical strength of C–C composites should be an integrated form of more variables than the porosity.

### 3.3. Derivation of the Optimal Factors to Maximize Mechanical Strength

In previous reports, the mechanical strength, S, of coke-based material follows the Knudsen equation [9,10]:

(3) $$S=S_{max}e^{-bp}$$

where S_max_ is the theoretical mechanical strength assuming a fully packed sample without pores, and p and b are the porosity and empirical constant, respectively. The Knudsen equation means that minimizing porosity is important to enhance the mechanical strength of C–C composites. Therefore, porosity can be used as an optimization factor for the coke-binder mixing ratio.

**Figure 5:**
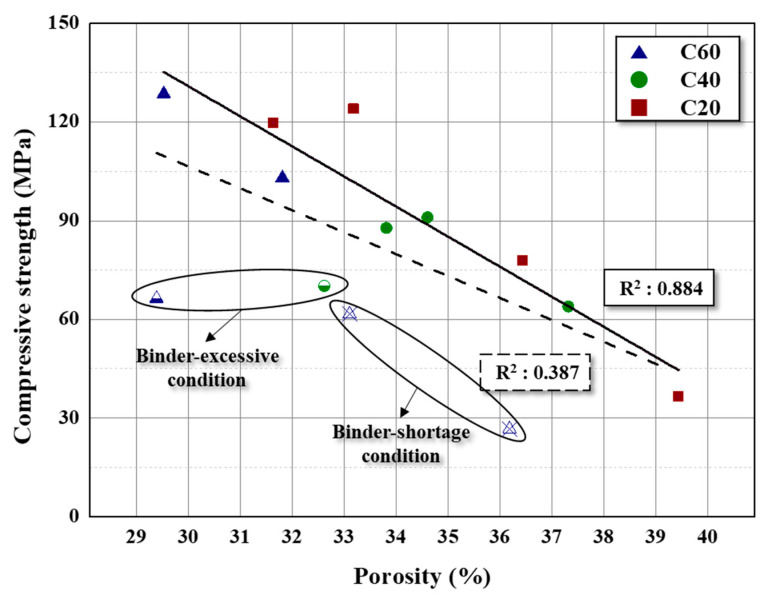
Correlation between the porosity and compressive strength of the prepared carbon blocks.

In this study, the correlation between the porosity and mechanical strength was analyzed, as shown in Figure 5. The R-squared correlation coefficient was 0.387, which shows a low correlation. However, the correlation coefficient increased to 0.884 (solid line) when the experimental groups that deviate from the linear relationship (marked as x and half symbols) were excluded. This tendency means that the optimization of the filler-and-binder mixing ratio using the porosity has limitations in the binder shortage and excessive conditions.

The data marked with the x symbol were prepared with the C60 filler using 10–15 vol.% binder mixing ratios. This condition is under the binder-shortage condition, whereby the resulting carbon blocks have high porosity. From the Knudsen equation, mechanical strength has an inversely proportional relationship with porosity. However, as the compressive strength decreased more rapidly, the trend deviates from the proportional relationship. This suggests that mechanical strength is affected by more factors, aside from the porosity. In particular, mechanical strength is also influenced by the adhesive strength. As the filler particles cannot attach perfectly under the binder-shortage conditions, the mechanical strength of the bulk block decreases. Consequently, the mechanical strength decreased more sharply than the proportional relationship of the binder-shortage conditions because the mechanical strength is affected by the porosity and attachment state.

The data marked as half symbols were prepared using the C60 and C40 filler with a 30 vol.% binder mixing ratio, which is under the binder-excessive condition, whereby the mechanical strength rapidly decreased, unlike the proportional relationship with the porosity, approaching 60 MPa. In particular, the block using C60 with 30 vol.% exhibits minimum porosity in all prepared samples. However, the mechanical strength decreased by half, from 128.61 MPa to 66.32 MPa (with 25 vol.% binder). In other words, carbonized excessive binder exists in the gap of the filler particles and does not influence the mechanical strength. Moreover, the thicker binder layer between the filler particles reduces the mechanical strength.

**Figure 6:**
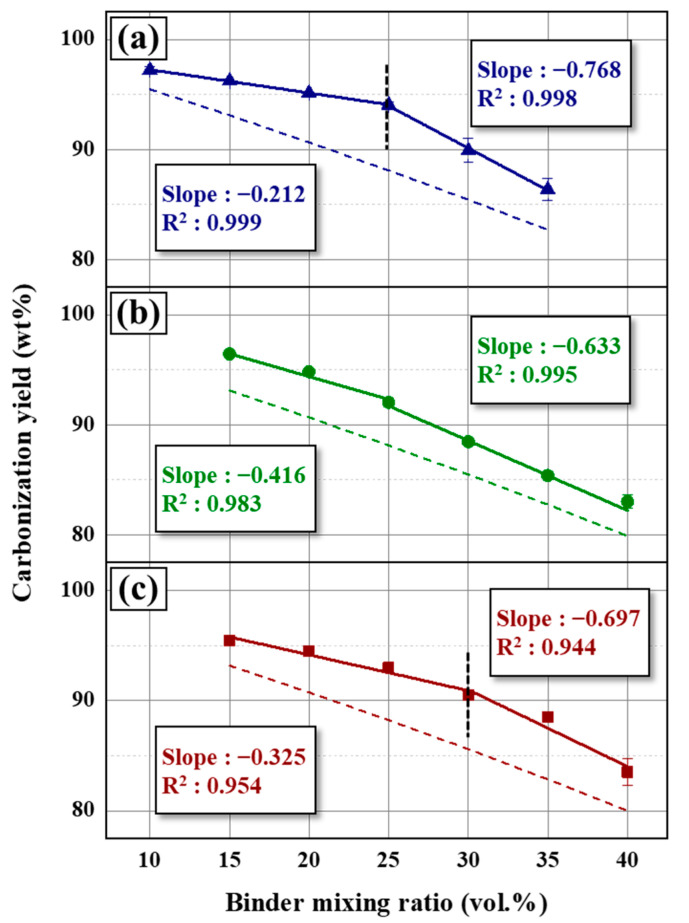
Effect of the binder mixing ratio on the carbonization yield of the prepared blocks using (**a**) C60, (**b**) C40, and (**c**) C20 as filler.

Along with the results in Section 3.2, it can be deduced that the tendency of the mechanical strength is divided into the binder-shortage and binder-excessive conditions, based on the optimum binder ratio. Moreover, blocks have different carbonization mechanisms between the two binder conditions, as shown in Figure 6. The inflection point is obtained from the trend of the compressive strength, as predicted by the carbonization tendency (solid line in Figure 6). In the C60 samples, the slope of the carbonization yield increased by 3.62, from −0.212 to −0.768, at a binder ratio of 25 vol.%. The inflection point shifted by 2.14 in the sample using C20 filler and a 30 vol.% binder ratio; however, the slope changing rate is lower than that at C60. This trend is consistent with the increased tolerance of the excess binder for smaller particles, corresponding to the compressive strength variations of the carbon blocks.

Based on the assumption that there is no interactive reaction between the filler and binder (dashed line in Figure 6), the carbonization yield of the binder pitch and filler coke was fixed at 34.30 and 100 wt.%, respectively. Therefore, the dashed line is only influenced by the binder ratio, whereby its slope constantly decreases with an increasing binder ratio. The gap between the solid and dashed line varies with the binder mixing ratio. The maximum gap was noted using the C60 filler with 25 vol.% binder and C20 filler with 30 vol.% binder. Under these conditions, the maximum mechanical strength was obtained, which indicates that the interaction between the coke and binder during carbonization is the main factor influencing the mechanical strength.

**Figure 7:**
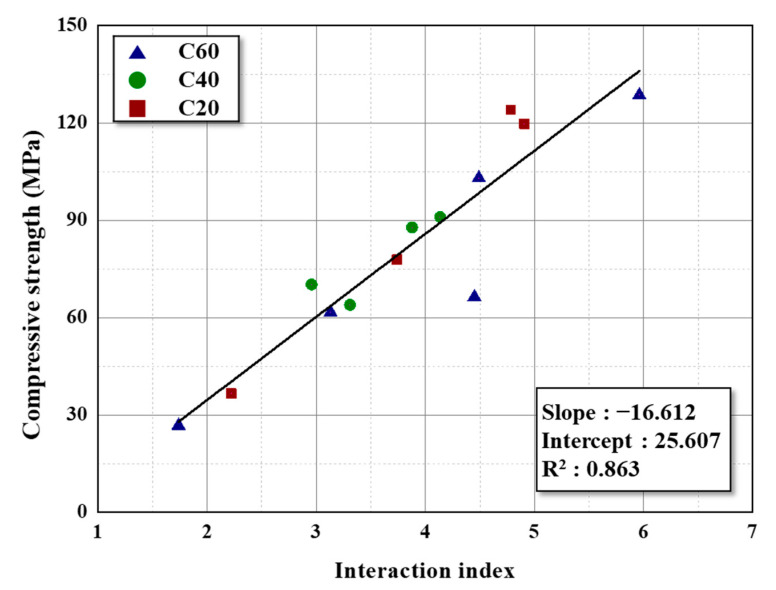
Correlation between the interaction index and compressive strength of the prepared carbon blocks with different fillers.

The interaction index, which represents the interaction between the filler and binder during carbonization, is shown in Figure 7. The interaction index was calculated by the difference between the carbonization yield of the blocks with (solid line in Figure 6) and without (dashed line in Figure 6) the interaction between the filler and binder during carbonization. The interaction index and compressive strength exhibit an excellent correlation with the R-squared correlation coefficient of 0.863 for all carbon blocks with different fillers.

In summary, the mechanical strength of the carbon block is mainly influenced by the interaction between the filler coke and binder pitch. The interaction index provides a quantification for the interaction between the coke and binder during carbonization, and can be used to optimize the binder mixing ratio of carbon blocks with high strength. Moreover, this parameter can predict the compressive strength of the carbon block.

## 4. Conclusions

The proportional relationship between porosity and mechanical strength in composites suggests that mechanical strength can be enhanced by decreasing the porosity. Therefore, this study optimized the binder ratio of carbon blocks to control porosity. However, the results of the proposed method are only applicable when the binder ratios are near the optimum condition, with the correlation coefficient decreasing from 0.884 to 0.387 as the binder ratio deviated from the optimum condition.

In this study, the effect of the binder ratio was considered under the binder-shortage and binder-excessive conditions. The binder pitch was considered responsible for two roles: the attachment between the surface of the filler particles and filling the void space between the filler particles. The effect of the void filling increased as the surface area of the particles was decreased, thereby decreasing the porosity with increasing filler particles in the binder-shortage condition. However, the filled binder has minimal effect on the mechanical strength. Thus, the correlation between the porosity and mechanical strength decreased under the binder-shortage condition. In contrast, a sufficient number of binders are available for the two roles under the binder-excessive condition. Thus, the mechanical strength is mainly influenced by the surface area of the filler particles, which offer a reaction site between the filler and binder. In summary, the mechanical strength of the blocks is influenced by the interaction reactivity between the filler and binder, which is referred to as the interaction index in this study.

The interaction index includes the effect of the particle size, specific surface area, surface roughness, pore structure, and SPAN of the particles. The interaction index exhibited a high correlation coefficient of 0.863 with the mechanical strength, regardless of the filler properties and binder ratios. Therefore, the interaction index can be applied in predicting the mechanical strength of carbon blocks and optimizing the binder mixing ratio for high-strength carbon blocks. Further, this parameter offers easy applicability in various industrial fields as it can be calculated using the carbonization yield without additional analysis.

## Figures and Tables

**Table 1:** Properties of the pitch and binder used in the study.

Properties		Binder Pitch	Pitch Coke
Softening point (°C)		103.4	–
Coking value (wt.%)		34.30	–
Aromaticity		1.097	–
Density (g/cm^3^)		1.322	–
Elemental contents	C (%)	92.35	98.94
	H (%)	4.35	0.28
	N (%)	0.84	0.67
	S (%)	0.68	0.11
	O (%)	1.78	–
	H/C ratio	0.57	–
Insoluble fraction	Hexane insoluble (wt.%)	65.14	–
	Toluene insoluble (wt.%)	27.65	–
	NMP insoluble (wt.%)	5.43	–
	α-resin (%)	5.43	–
	β-resin (%)	22.22	–
	γ-resin (%)	72.35	–

**Table 2:** Characteristics of the particles of the prepared cokes.

Sample	Particle Size (d_50_, μm)	SPAN	True Density (g/cm^3^)	Tap Density (g/cm^3^)	Specific Surface Area (m^2^/g)
C60	62.13	0.76	1.995	1.16	1.17
C40	46.44	0.74	2.001	1.12	1.66
C20	27.10	0.90	2.015	1.04	2.32

## Data Availability

Data are contained within the article.

## Supplementary Materials

The following supporting information can be downloaded at: https://www.mdpi.com/article/10.3390/ma16114084/s1, Figure S1: XRD pattern of regular coke used as the filler; Table S1: Mixing ratio of the filler coke and binder pitch to prepare the carbon blocks; Figure S2: Nitrogen adsorption-desorption isotherms of the prepared coke particles using (a) C60, (b) C40, and (c) C20 fillers; Figure S3: Carbon block and mold in an unformed condition; (a) Cracked block based with insufficient binder, (b) Binder leaking from the gaps of the molds with excessive binder.
