# Supplementary material for: Optimization of the Filler-and-Binder Mixing Ratio for Enhanced Mechanical Strength of Carbon–Carbon Composites
Source: Materials (Basel). 2023 May 30;16(11):4084. doi: 10.3390/ma16114084 (PMC10254209; doi:10.3390/ma16114084)
Supplement: Supplementary file 1 [file materials-16-04084-s001.zip › materials-2365616-supplementary.pdf]

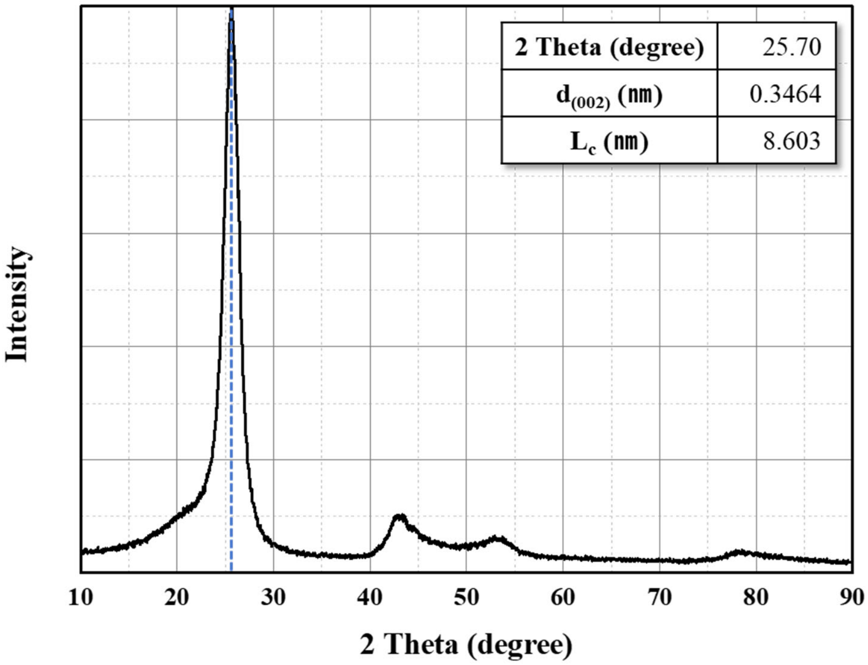

Figure S1. XRD pattern of regular coke used as the filler.

Table S1. Mixing ratio of the filler coke and binder pitch to prepare the carbon blocks.

Binder mixing ratio (vol.%)	C60		C40		C20	
	Coke (g)	Pitch (g)	Coke (g)	Pitch (g)	Coke (g)	Pitch (g)
10	9.3138	0.6862	9.3158	0.6842	–	–
15	8.9525	1.0475	8.9554	1.0446	8.9622	1.0378
20	8.5781	1.4219	8.5819	1.4181	8.5907	1.4093
25	8.1899	1.8101	8.1946	1.8054	8.2052	1.7948
30	7.7872	2.2128	7.7926	2.2074	7.8050	2.1950
35	7.3690	2.6310	7.3752	2.6248	7.3891	2.6109
40	–	–	6.9414	3.0586	6.9567	3.0433
45	–	–	–	–	6.5066	3.4934

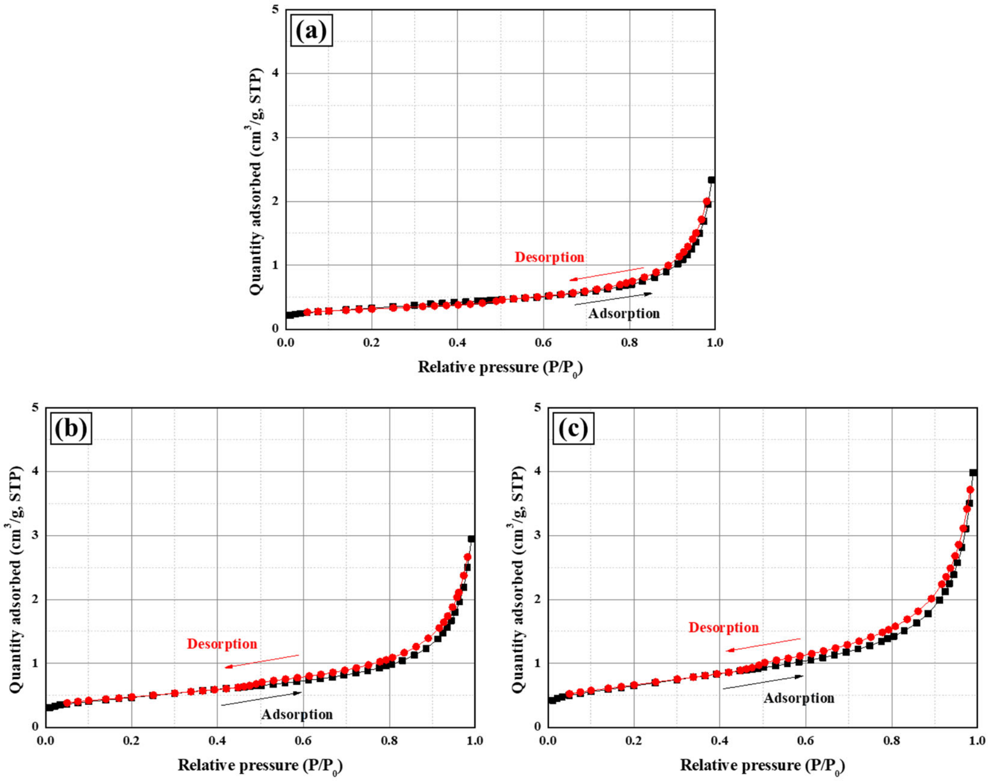

Figure S2. Nitrogen adsorption–desorption isotherms of the prepared coke particles using (**a**) C60, (**b**) C40, and (**c**) C20 fillers.

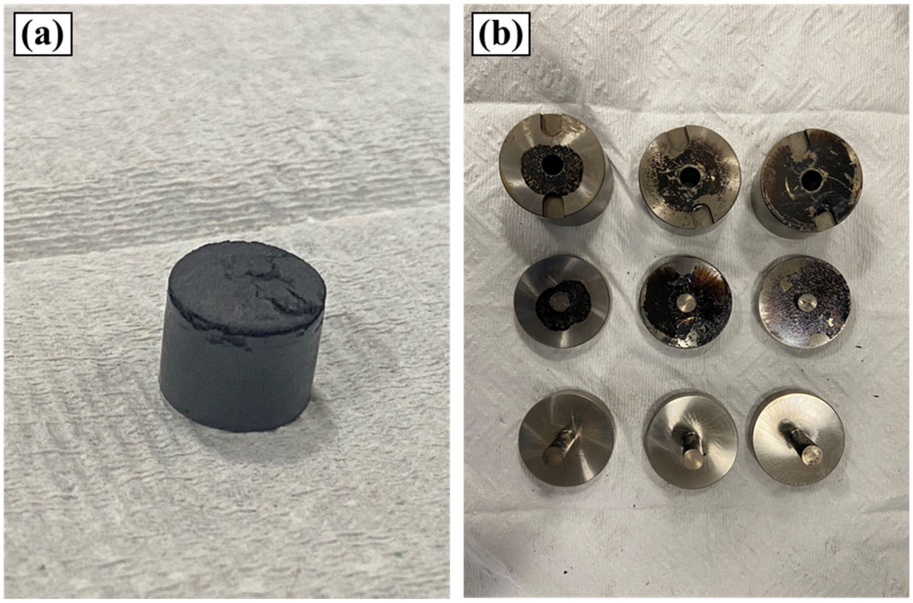

Figure S3. Carbon block and mold in an unformed condition; (**a**) Cracked block based with insufficient binder, (**b**) Binder leaking from the gaps of the molds with excessive binder.
